# Increased liver glycogen levels enhance exercise capacity in mice

**DOI:** 10.1016/j.jbc.2021.100976

**Published:** 2021-07-18

**Authors:** Iliana López-Soldado, Joan J. Guinovart, Jordi Duran

**Affiliations:** 1 Institute for Research in Biomedicine (IRB Barcelona), The Barcelona Institute of Science and Technology, Barcelona, Spain; 2 Centro de Investigación Biomédica en Red de Diabetes y Enfermedades Metabólicas Asociadas (CIBERDEM), Madrid, Spain; 3 Department of Biochemistry and Molecular Biomedicine, University of Barcelona, Barcelona, Spain

**Keywords:** exercise, glycogen, glucose, protein targeting to glycogen (PTG), liver metabolism, ATP, FFAs, free fatty acids, G6P, glucose-6-phosphate, *G6pase*, glucose-6-phosphatase, GS, glycogen synthase, *Nr4a3*, NR4A member 3, *Pdk4*, pyruvate dehydrogenase kinase 4, PEPCK, phosphoenolpyruvate carboxykinase, *Pgc1α*, peroxisome proliferator-activated receptor gamma coactivator 1-alpha, PTG, protein targeting to glycogen, PTG^OE^, overexpressing PTG specifically in the liver, TAGs, triacylglycerols

## Abstract

Muscle glycogen depletion has been proposed as one of the main causes of fatigue during exercise. However, few studies have addressed the contribution of liver glycogen to exercise performance. Using a low-intensity running protocol, here, we analyzed exercise capacity in mice overexpressing protein targeting to glycogen (PTG) specifically in the liver (PTG^OE^ mice), which show a high concentration of glycogen in this organ. PTG^OE^ mice showed improved exercise capacity, as determined by the distance covered and time ran in an extenuating endurance exercise, compared with control mice. Moreover, fasting decreased exercise capacity in control mice but not in PTG^OE^ mice. After exercise, liver glycogen stores were totally depleted in control mice, but PTG^OE^ mice maintained significant glycogen levels even in fasting conditions. In addition, PTG^OE^ mice displayed an increased hepatic energy state after exercise compared with control mice. Exercise caused a reduction in the blood glucose concentration in control mice that was less pronounced in PTG^OE^ mice. No changes were found in the levels of blood lactate, plasma free fatty acids, or β-hydroxybutyrate. Plasma glucagon was elevated after exercise in control mice, but not in PTG^OE^ mice. Exercise-induced changes in skeletal muscle were similar in both genotypes. These results identify hepatic glycogen as a key regulator of endurance capacity in mice, an effect that may be exerted through the maintenance of blood glucose levels.

Glycogen is the storage form of glucose in mammals. Glycogen synthesis is catalyzed by glycogen synthase (GS). There are two mammalian isoforms of GS. One, encoded by the *Gys2* gene, is expressed only in the liver (1), whereas a second gene, *Gys1*, is expressed in the skeletal muscle, cardiac muscle, adipose tissue, kidneys, and brain (2). GS is regulated by covalent phosphorylation (3) and allosteric effectors (4). Dephosphorylation and thus activation of GS is catalyzed by protein phosphatase 1. A family of scaffolding proteins target the protein phosphatase 1 catalytic subunit to glycogen particles, where the enzymes of glycogen metabolism are concentrated. There are seven scaffolding proteins, among them the ubiquitously distributed protein targeting to glycogen (PTG). Overexpression of PTG has been used to activate GS and thus promote glycogen synthesis (5, 6, 7, 8, 9, 10).

Carbohydrate and fat are the main substrates used during prolonged endurance-type exercise (11, 12, 13). The relative contribution of each is determined primarily by the intensity and duration of exercise (11, 12). During moderate- to high-intensity exercise, carbohydrate is the main source of substrate. Given that carbohydrate stores in the form of glycogen (primarily in the liver and muscle) are relatively small, endurance-type exercise performance/capacity is often limited by endogenous carbohydrate availability (13).

The importance of muscle glycogen availability during prolonged exercise has received much attention over the last 50 years (13, 14, 15), and a strong association has been reported between muscle glycogen depletion, impaired muscle performance, and fatigue (16, 17, 18, 19, 20, 21). In contrast, little attention has been paid to the contribution of liver glycogen during exercise. Liver glycogen stores are mobilized during exercise in response to the increased glucose demands of contracting skeletal muscle (22, 23, 24). Accordingly, continuous exercise lasting >60 min in humans substantially depletes liver glycogen (25). Interestingly, in humans, liver glycogen sparing in an endurance-trained state may account partly for training-induced performance/capacity adaptations during prolonged (>90 min) exercise (13). Liver GS KO mice, which have a 95% reduction in the liver glycogen content, have a decreased capacity for exhaustive high-intensity running compared with control mice. This difference disappears after an overnight fast, which reduces the liver glycogen of control mice to levels comparable with those of *Gys2* KO animals (26). These results indicate that the lack of liver glycogen impairs endurance capacity. We sought to analyze endurance capacity in animals overexpressing PTG specifically in the liver (PTG^OE^ mice) and that maintain relatively high levels of hepatic glycogen even after fasting. To extrapolate our results to endurance sports such as marathon running or cycling, a low-intensity running protocol was used. We show that PTG^OE^ mice indeed have a greater endurance capacity than control animals. Importantly, fasting did not significantly reduce the capacity of PTG^OE^ mice. All together, these results highlight the role of liver glycogen in resistance to fatigue.

## Results

### Generation of mice with liver-specific PTG overexpression

**Figure 1:**
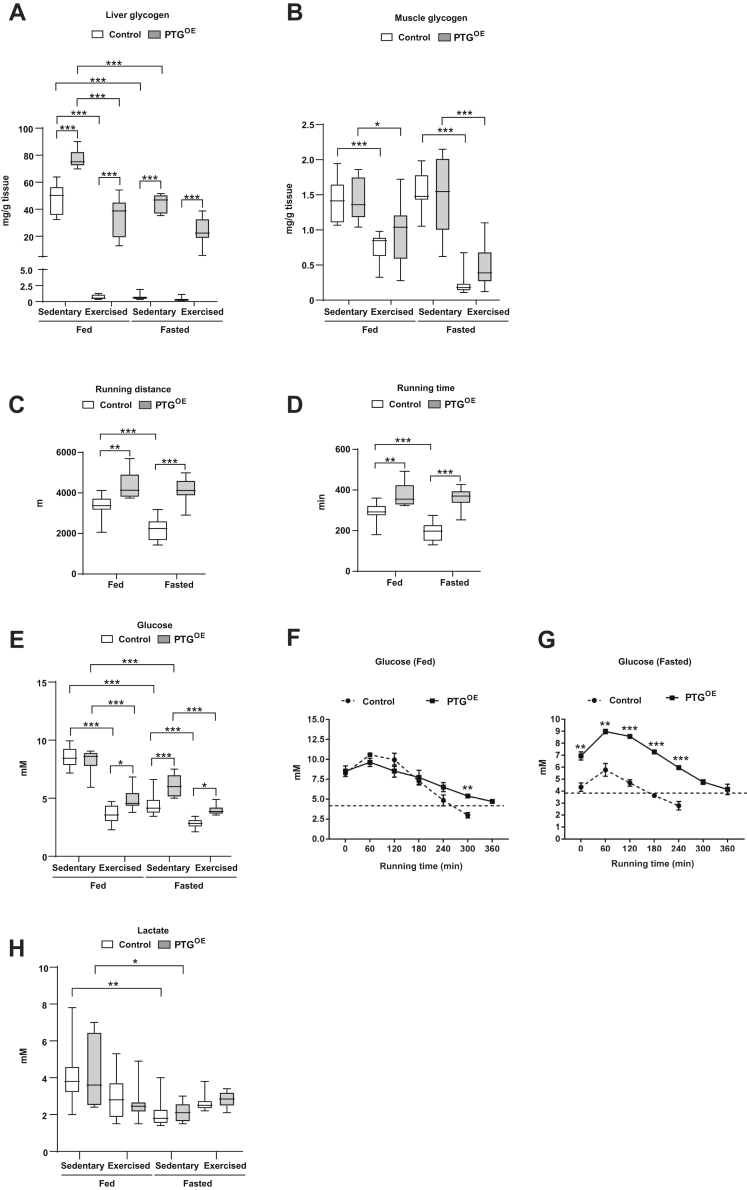
**PTG**^**OE**^**mice showed enhanced endurance exercise capacity.***A*, liver glycogen, (*B*) muscle glycogen, (*C*) running distance, (*D*) running time, (*E*–*G*) blood glucose, and (*H*) blood lactate in sedentary and exercised control and PTG^OE^ mice under fed and fasting conditions (n = 8–12 in experiments *A*–*E* and *H*; n = 3 in experiments *F* and *G*). All values are the mean ± SEM. ∗*p* < 0.05, ∗∗*p* < 0.01, and ∗∗∗*p* < 0.001. PTG, protein targeting to glycogen; PTG^OE^, overexpressing PTG specifically in the liver.

To study the role of increased liver glycogen storage in endurance capacity, we generated PTG^OE^ mice (see Experimental procedures). The mRNA level of *Ptg* in the livers of these mice was 8-fold greater than that of control animals (Fig. S1*A*). PTG^OE^ mice showed an increase in hepatic glycogen compared with control mice both in fed and fasting conditions (Fig. 1*A*), as previously described (27). There was no difference in the glycogen content of muscles (Fig. 1*B*). Before the exercise experiments, the body weight was equivalent between control and PTG^OE^ mice. After overnight fasting, the weight of the animals of both genotypes was similarly diminished compared with fed mice (Fig. S2).

### PTG^OE^ mice showed enhanced endurance exercise capacity

To assess capacity for endurance exercise, control and PTG^OE^ mice were forced to run to exhaustion under fed and fasting conditions. PTG^OE^ mice ran significantly more (≈30% of the distance and of the time ran) than control mice both in fed and fasting conditions (Fig. 1, *C* and *D*). Interestingly, fasting induced a decrease (≈35% of the distance and of the time ran) for endurance exercise capacity in control mice but not in PTG^OE^ animals (Fig. 1, *C* and *D*). Upon completion of the exercise, liver glycogen stores were totally depleted in fed control mice but not in fed PTG^OE^ ones, which were able to maintain liver glycogen at 30 mg/g of tissue (Fig. 1*A*). Strikingly, glycogen stores were not depleted in fasted PTG^OE^ mouse livers even after exhaustive exercise (Fig. 1*A*). Muscle glycogen was depleted similarly in both genotypes under fed and fasting conditions (Fig. 1*B*).

It has been suggested that a decrease in systemic glucose limits endurance exercise (28). In our experiments, basal blood glucose was similar between genotypes under fed conditions. However, fasting and exercise caused a reduction in blood glucose concentration in control mice, and this reduction was less pronounced in PTG^OE^ mice (Fig. 1*E*). We also monitored blood glucose every hour during the run-to-exhaustion test. Glucose levels were maintained above 3.9 mM (70 mg/dl) for longer in PTG^OE^ mice than control animals in both fed (Fig. 1*F*) and fasting conditions (Fig. 1*G*). In fact, in fasting conditions, glucose levels were higher in PTG^OE^ mice throughout the test. Lactate is a gluconeogenic precursor released from skeletal muscle during exercise and taken up by the liver to be converted to pyruvate and used in gluconeogenesis. Blood lactate was similar between the two genotypes in all conditions (Fig. 1*H*).

### Liver and muscle metabolites

**Figure 2:**
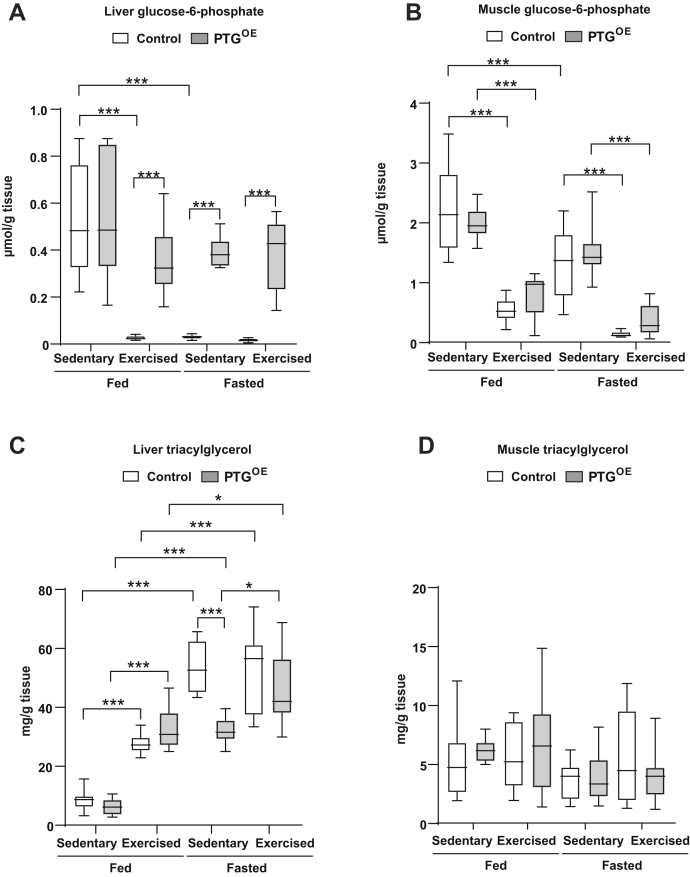
**Liver and muscle metabolites.***A*, liver glucose-6-phosphate, (*B*) muscle glucose-6-phosphate, (*C*) liver triacylglycerol, and (*D*) muscle triacylglycerol in sedentary and exercised control and PTG^OE^ mice under fed and fasting conditions (n = 8–12 in all experiments). All values are the mean ± SEM. ∗*p* < 0.05 and ∗∗∗*p* < 0.001. PTG, protein targeting to glycogen; PTG^OE^, overexpressing PTG specifically in the liver.

It has been described that the levels of G6P in the liver decrease immediately after exercise (29). Liver G6P was almost undetectable after exercise and/or fasting in control mice. In contrast, PTG^OE^ mice maintained relatively high levels of G6P, about 65% of that observed in control fed animals, after exercise and also in fasting conditions (Fig. 2*A*). In the muscle, G6P was similarly depleted after exercise in both genotypes under fed and fasting conditions (Fig. 2*B*).

Hepatic TAGs acutely accumulate during and after exercise in rodents (30). We found that exercise and fasting increased hepatic TAG concentration in fed control and PTG^OE^ mice (Fig. 2*C*). Fasted PTG^OE^ mice showed lower liver TAG levels than control mice before exercise, but after exercise, the concentration of TAG was similar in the two genotypes (Fig. 2*C*). Exercise did not significantly alter TAG concentration in muscle (Fig. 2*D*).

### Plasma hormones and metabolites

**Figure 3:**
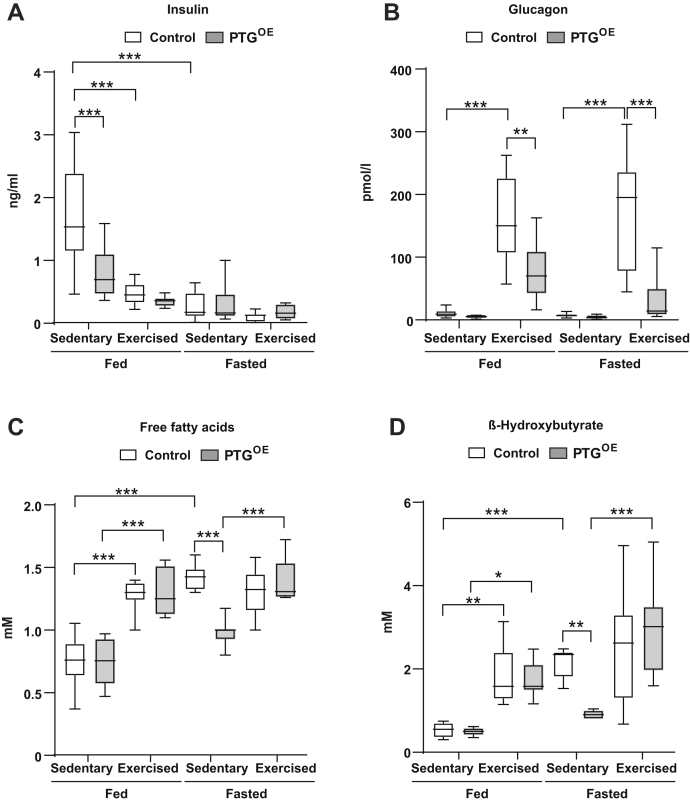
**Plasma hormones and metabolites.***A*, plasma insulin, (*B*) plasma glucagon, (*C*) plasma free fatty acids, and (*D*) plasma β-hydroxybutyrate in sedentary and exercised control and PTG^OE^ mice under fed and fasting conditions (n = 8–12 in all experiments). All values are the mean ± SEM. ∗*p* < 0.05, ∗∗*p* < 0.01, and ∗∗∗*p* < 0.001. PTG, protein targeting to glycogen; PTG^OE^, overexpressing PTG specifically in the liver.

Exercise is characterized by complex endocrine responses (31). If exercise is sustained, a decrease in insulin secretion and increases in glucagon are observed (32). In fed control mice, plasma insulin was significantly reduced after exercise (Fig. 3*A*). Insulin concentration was already lower in resting PTG^OE^ mice, as previously described (27) and it was not further reduced after exercise. Glucagon increased dramatically after exercise in both genotypes, but this increment was lower in PTG^OE^ mice under fed and fasting conditions (Fig. 3*B*).

During exercise, FFAs and β-hydroxybutyrate become a major fuel source for other organs (33, 34). Under fed conditions, exercise increased plasma FFAs and β-hydroxybutyrate levels similarly in both genotypes (Fig. 3, *C* and *D*). In fasted conditions, plasma FFAs (Fig. 3*C*) and β-hydroxybutyrate (Fig. 3*D*) were lower in sedentary PTG^OE^ than control mice as we previously described (27). However, exercise increased the levels of FFAs and β-hydroxybutyrate to similar levels in both genotypes (Fig. 3, *C* and *D*).

### Liver and muscle nucleotides

**Figure 4:**
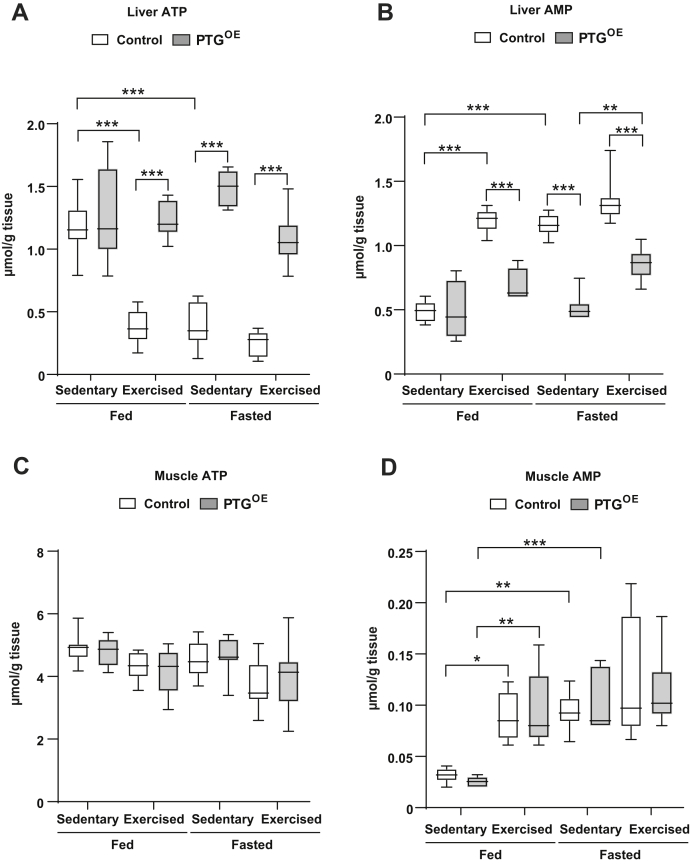
**Liver and muscle nucleotides.***A*, liver ATP, (*B*) liver AMP, (*C*) muscle ATP, and (*D*) muscle AMP in sedentary and exercised control and PTG^OE^ mice under fed and fasting conditions (n = 8–12 in all experiments). All values are the mean ± SEM. ∗*p* < 0.05, ∗∗*p* < 0.01, and ∗∗∗*p* < 0.001. PTG, protein targeting to glycogen; PTG^OE^, overexpressing PTG specifically in the liver.

Exercise causes a drop in hepatic ATP (31). Our results showed that liver ATP concentration decreased and AMP concentration increased in control mice after exercise (Fig. 4, *A* and *B*). Fasted control mice also showed lower ATP and higher AMP levels than fed control counterparts (Fig. 4, *A* and *B*), as previously shown (35). Remarkably, the ATP and AMP levels of exercised fed and fasted PTG^OE^ mice were maintained at similar levels to those of fed sedentary mice. The energy state of muscle is less sensitive to changes in metabolic demand than that of the liver (36). Therefore, muscle ATP levels were similar in all the groups (Fig. 4*C*). However, AMP concentration was significantly increased after exercise and fasting similarly in both genotypes (Fig. 4*D*).

### Liver metabolism enzymes

**Figure 5:**
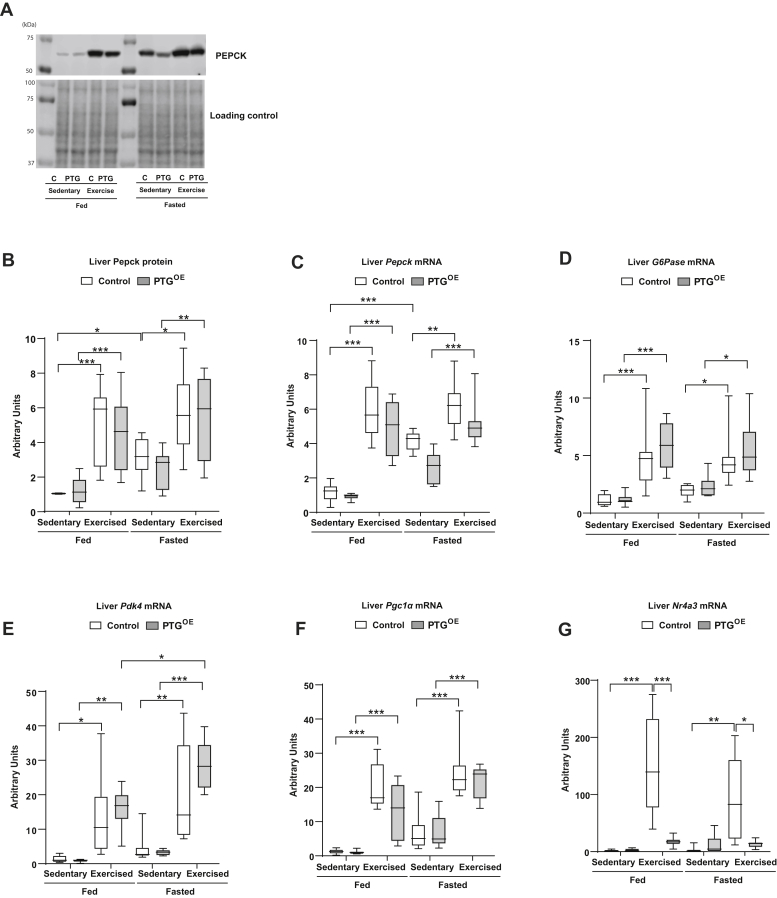
**Liver metabolism enzymes.***A*, representative Western blot image of Pepck and loading control, (*B*) Pepck protein expression, (*C*) *Pepck* mRNA expression, (*D*) *G6pase* mRNA expression, (*E*) *Pdk4* mRNA expression, (*F*) *Pgc1α* mRNA expression, and (*G*) *Nr4a3* mRNA expression in sedentary and exercised control and PTG^OE^ mice under fed and fasting conditions (n = 8–12 in all experiments). All values are the mean ± SEM. ∗*p* < 0.05, ∗∗*p* < 0.01, and ∗∗∗*p* < 0.001. *G6pase*, glucose-6-phosphatase; *Nr4a3*, NR4A member 3; *Pdk4*, pyruvate dehydrogenase kinase 4; PEPCK, phosphoenolpyruvate carboxykinase; *Pgc1α*, peroxisome proliferator-activated receptor gamma coactivator 1-alpha; PTG, protein targeting to glycogen; PTG^OE^, overexpressing PTG specifically in the liver.

The liver has to adapt to an enormous metabolic challenge during exercise. We studied the expression of enzymes known to be regulated by exercise in this organ (37). The protein level of Pepck was higher at exhaustion in control and PTG^OE^ mice under fed and fasting conditions (Fig. 5, *A* and *B*). The mRNA expression of *Pepck* and glucose-6-phosphatase (*G6pase*) was similarly upregulated in control and PTG^OE^ mice after exercise (Fig. 5, *C* and *D*). Pyruvate dehydrogenase kinase 4 (*Pdk4*) phosphorylates and inactivates the pyruvate dehydrogenase complex, thus inhibiting glucose oxidation and promoting gluconeogenesis (38). *Pdk4* mRNA expression was also similarly upregulated in exercised control and PTG^OE^ mice (Fig. 5*E*). Peroxisome proliferator-activated receptor gamma coactivator 1-alpha (*Pgc1α*) has been identified as an important regulator of hepatic gluconeogenesis (39). The mRNA expression of *Pgc1α* was increased in exercised mice compared with sedentary mice, but there were no changes between the genotypes (Fig. 5*F*). NR4A orphan nuclear receptors are induced by multiple extracellular signals and hormones in a cell type–specific manner (40, 41). In the liver, NR4A receptors are transcriptional modulators of hepatic glucose metabolism. Interestingly, we found that hepatic gene expression of NR4A member 3 (*Nr4a3*) was increased in control exercise mice but not in PTG^OE^ mice (Fig. 5*G*). We also measured the hepatic expression of genes implicated in glycogen metabolism. The expression of *Gys2* was upregulated in control but not in PTG^OE^ mice after exercise and fasting (Fig. S1*B*). Glycogen phosphorylase was downregulated after fasting in control but not in PTG^OE^ mice (Fig. S1*C*). Glycogen branching enzyme (*Gbe-1*) and phosphoglucomutase (*Pgm2*) were upregulated after fasting in control mice but not in PTG^OE^ mice (Fig. S1, *D* and *E*). Sedentary fed PTG^OE^ animals showed a lower glucokinase (*Gk*) mRNA expression than control mice (Fig. S1*F*), probably as a consequence of hypoinsulinemia. Moreover, *Gk* gene expression was downregulated after exercise in control but not in PTG^OE^ mice under fed conditions (Fig. S1*F*).

### Muscle metabolism enzymes

**Figure 6:**
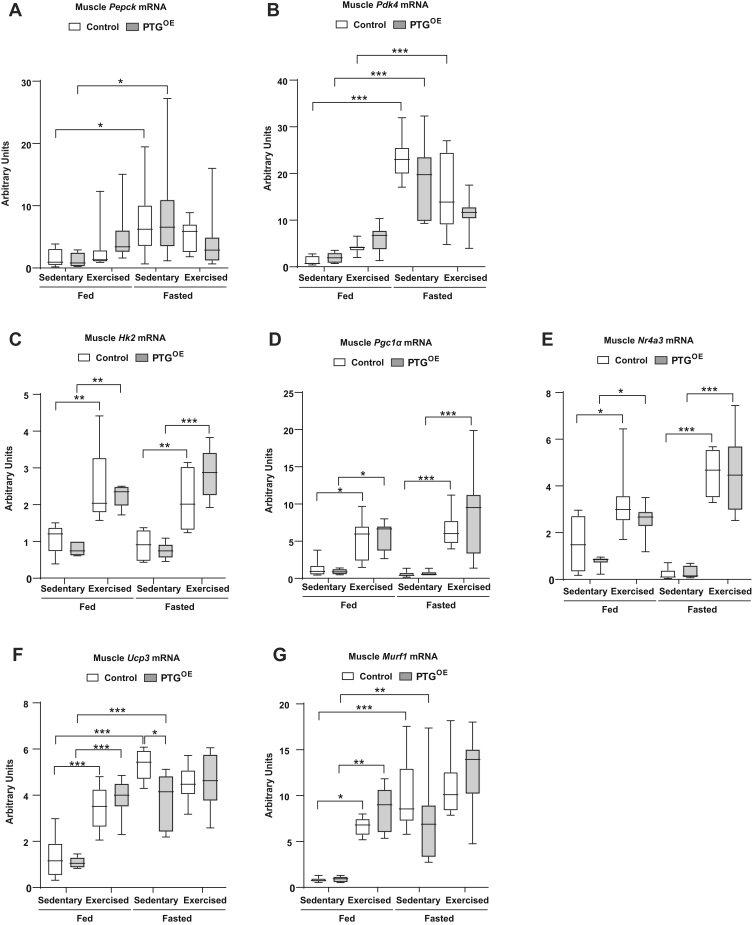
**Muscle gene expression.***A*, *Pepck* mRNA expression, (*B*) Pdk4 mRNA expression, (*C*) Hk2 mRNA expression, (*D*) *Pgc1α* mRNA expression, (*E*) *Nr4a3* mRNA expression, (*F*) *Ucp3* mRNA expression, and (*G*) *Murf1* mRNA expression in sedentary and exercised control and PTG^OE^ mice under fed and fasting conditions (n = 8–12 in all experiments). All values are the mean ± SEM. ∗*p* < 0.05, ∗∗*p* < 0.01, and ∗∗∗*p* < 0.001. Hk2, hexokinase-2; *Murf1*, RING-finger protein-1; *Nr4a3*, NR4A member 3; *Pdk4*, pyruvate dehydrogenase kinase 4; PEPCK, phosphoenolpyruvate carboxykinase; *Pgc1α*, peroxisome proliferator-activated receptor gamma coactivator 1-alpha; PTG, protein targeting to glycogen; PTG^OE^, overexpressing PTG specifically in the liver; *Ucp3*, uncoupling protein 3.

Exercise is associated with profound changes in skeletal muscle. We sought to determine whether the increased exercise capacity observed in PTG^OE^ mice is associated with changes in some of the main genes related to muscle metabolism. In muscle, fasting, but not exercise, induced the expression of *Pepck* (Fig. 6*A*) and *Pdk4* (Fig. 6*B*) in both genotypes. Exercise induced an increase in the expression of hexokinase-2 (Fig. 6*C*), *Pgc1α* (Fig. 6*D*), and *Nr4a3* (Fig. 6*E*) in muscle under fed and fasting conditions, but there were no differences between the genotypes. Exercise upregulated the expression of uncoupling protein 3 (Fig. 6*F*) and muscle RING-finger protein-1 (Fig. 6*G*) under fed but not under fasting conditions because fasting already induced an increase in these genes.

## Discussion

Many mechanisms may be involved in the occurrence of fatigue, including changes in the internal environment (blood, extracellular fluid), changes within muscle fibers, and effects on the central nervous system (central fatigue) (42). It has been proposed that depletion of liver and muscle glycogen leads to exhaustion (13, 16). The importance of muscle glycogen for performance was subsequently confirmed in numerous studies in humans (43, 44, 45). However, studies addressing the contribution of liver glycogen to exercise capacity are limited in humans presumably because of the methodological limitations when attempting to assess liver glycogen content in these subjects (13). In an effort to elucidate the role of hepatic glycogen in endurance capacity, we generated PTG^OE^ mice with a high concentration of hepatic glycogen. Using a low-intensity running protocol, we found that these mice were able to run for greater distances and longer, thereby indicating that liver glycogen is strongly associated with endurance capacity. Fasting completely depleted liver glycogen in control mice, and fasted animals ran significantly less than fed mice, as previously described (26). In contrast, PTG^OE^ mice maintained sizeable concentrations of hepatic glycogen even after fasting and showed a similar endurance capacity to fed animals. These results confirm a key role of liver glycogen in exercise performance in mice.

In this study, we show that the key factor by which increased liver glycogen stores contributes to endurance capacity is the preservation of blood glucose. Hepatic glucose output is the primary source of the increased glucose available to exercising muscle (46). During prolonged exercise, glucose utilization by working muscle may outstrip glucose production, resulting in the gradual development of hypoglycemia (46). Several studies show that the strongest correlation to endurance is the maintenance of blood glucose above 3.9 mM (70 mg/dl) (28, 47). We found an increase in blood glucose after 1 h of exercise and a gradual decline during the running test in both genotypes, but blood glucose declined more slowly in PTG^OE^ mice. Fed control mice showed hypoglycemia (blood glucose levels <3.9 mM) at the time of exhaustion, that is, after 5 h of running in fed conditions or after 3 h of running in fasting conditions. In contrast, PTG^OE^ mice maintained a blood glucose concentration above 3.9 mM throughout the test in both conditions, probably because of higher glycogenolysis, in keeping with higher glycogen stores, which is reflected in the maintenance of substantial levels of G6P even at exhaustion. Moreover, the fall in blood glucose during prolonged exercise is accompanied by a reduction in insulin (48) and a rise in glucagon concentrations (49), particularly if a degree of hypoglycemia ensues. PTG^OE^ mice showed a lower glucagon concentration than control mice after running under fed and fasting conditions. This observation is in line with the absence of hypoglycemia in these mice. Moreover, plasma insulin concentration under fed condition was lower in sedentary PTG^OE^ mice than control mice (27, 35). This observation could be explained by the improved glucose tolerance observed in these mice (27). Furthermore, the levels of insulin were not reduced after exercise in PTG^OE^ mice.

Gluconeogenesis is important for maintaining blood glucose levels during exercise (50), and this process is regulated at the transcriptional level by key enzymes, namely *Pepck* and *G6pase*, which are increased during exercise (51). At exhaustion, the hepatic expression of *Pepck*, *G6pase*, *Pdk4*, and *Pgc1α* was equally upregulated in fed control and PTG^OE^ mice, thereby indicating that gluconeogenesis was similarly increased in the livers of both groups of mice. Thus, the ability of PTG^OE^ mice to preserve glucose levels during exercise was not the result of an increased expression of these genes.

The NR4A family of nuclear hormone receptors has been shown to regulate varied processes across a host of tissues (52). *Nr4a3*, also known as *NOR1*, has been identified as one of the most exercise responsive genes in the skeletal muscle of humans (53), but the role of hepatic *Nr4a3* in exercise has not been explored. Interestingly, exercise induced the expression of hepatic *Nr4a3* in control but not in PTG^OE^ mice, which could be explained by the circulating levels of glucagon. Indeed, the hepatic expression of *Nr4a3* is potently induced by glucagon *in vivo* in a cAMP-dependent manner (54), and PTG^OE^ mice showed lower plasma glucagon at exhaustion than control mice.

During exercise, adipose tissue lipolysis is stimulated by hormonal changes, resulting in increased availability and tissue utilization of FFAs. In the circulation, the concentration of β-hydroxybutyrate increased after exercise, indicating an acceleration of ketogenesis. Plasma FFAs and β-hydroxybutyrate were similar in both genotypes after exercise, indicating that differences in adipose tissue lipolysis did not affect endurance capacity. Fasting and exercise in mice induced hepatic steatosis as a result of excessive uptake of circulating FFAs derived from adipose tissue lipolysis. The amount of hepatic TAG content was lower in fasted PTG^OE^ mice than control mice, which could be attributable to a lower flux of FFAs reaching the liver, as previously described (27). However, hepatic steatosis was present to a similar extent in exercised control and PTG^OE^ mice, and this finding correlates with the amount of circulating FFAs detected.

Exercise results in a deficit in the energy status of the liver as defined by increased hepatic AMP concentrations and decreased ATP concentrations (36). Strikingly, exercised PTG^OE^ mice maintained ATP and AMP at the same level as sedentary counterparts, both in fed and fasting conditions. Thus, increasing liver glycogen maintained hepatic energy status during exercise. In this regard, we previously demonstrated that liver glycogen maintains hepatic energy state during fasting (35) and diabetes (27, 55, 56). In mice, metabolic stress and a physiological rise in glucagon caused a decrease in the hepatic energy state (57). PTG^OE^ mice did not show higher levels of glucagon, which would explain the maintenance of hepatic energy status.

A strong association has been suggested between muscle glycogen depletion, impaired muscle performance, and fatigue development during exercise (16, 17). Thus, we analyzed whether the increased exercise capacity observed in PTG^OE^ mice is associated with changes in skeletal muscle. We found that muscle glycogen, G6P, TAG, ATP, and AMP content were similarly changed in both genotypes after exercise. This observation thus indicates that these metabolites did not have an effect on endurance capacity in this model. Plasma lactate, an indicator for muscle glycolysis, was similar in both genotypes, thus indicating that lactate was not related to the increased performance in PTG^OE^ mice. We also analyzed genes whose gain of function in skeletal muscle increases endurance performance in mice (58). The expression of *Pepck*, hexokinase-2, *Pgc1α*, *Nr4a3*, uncoupling protein 3, and RING-finger protein-1 in skeletal muscle was similar in both genotypes, thus indicating that they were not involved in the increased performance of the PTG^OE^ mice.

In conclusion, these results identify hepatic glycogen as a key regulator of endurance capacity in mice, an effect that may be exerted through the maintenance of blood glucose. Thus, in endurance sports such as marathon running and long-distance cycling, increasing liver glycogen stores could maintain blood glucose and delay the onset of hypoglycemia or “hitting the wall.”

## Experimental procedures

### Animals

All procedures were approved by the Barcelona Science Park Animal Experimentation Committee and carried out in accordance with the European Community Council Directive and the National Institutes of Health guidelines for the care and use of laboratory animals. PTG^OE^ mice were generated as previously described (27). Briefly, the PTG cDNA under the control of the ubiquitous CAG promoter (cytomegalovirus immediate early enhancer/chicken b-actin promoter fusion) was introduced into an innocuous locus by homologous recombination. A loxP-flanked transcription stop cassette was included between the CAG promoter and the PTG cDNA to allow the expression to depend on the action of a Cre recombinase. The resulting mouse line was bred with an albumin promoter Cre recombinase–expressing animal (The Jackson Laboratory), which drove the expression of PTG specifically to the liver. The PTG^OE^ mice were backcrossed to C57BL/6. Studies were performed in 5-month-old male mice. *Ad libitum*–fed animals or, when indicated, 16-h overnight-fasted animals were used. Fed and fasted mice were enrolled in the exercise protocol at 8.00 AM. For fasted mice, food was removed at 4.00 PM on the day before the experiment, and animals were fasted for 16 h until the next morning at 8.00 AM when the experimental runs started. Immediately after reaching exhaustion, the mice were sacrificed by cervical dislocation, and the tissues (liver and muscle) were collected in liquid nitrogen and stored at −80 °C for further analysis. Littermates of the same age were used as controls.

### Exercise protocol

A motorized, speed-controlled treadmill was used to exercise the mice (LE8710; Harvard Apparatus). Before running until exhaustion, mice were preadapted to the treadmill for four consecutive days (3 min/day). For the experimental run, mice ran at 8.00 AM on a treadmill set at a 20° incline with an initial belt speed of 12 cm/s. The speed was increased by 1 cm/s at 2, 5, 10, 20, 30, 40, 50, and 60 min after initiation of the exercise, followed by the exhaustion run at 20 cm/s until mice failed.

### Blood metabolite and hormone levels

Whole-blood samples were collected from the tail into EDTA-microvettes (Sarstedt Inc) and were then centrifuged at 1000*g* for 10 min at 4 °C. Supernatants were aliquoted and stored at −20 °C. Plasma insulin and glucagon were determined by ELISA (Crystal Chem). Plasma free fatty acids (FFAs) (Abcam) and β-hydroxybutyrate (Sigma-Aldrich) were measured using a commercial kit.

Blood glucose and lactate levels were measured using a glucometer (Bayer Contour Next, Bayer Healthcare) and a lactometer (Lactate Scout 4 analyzer, EKF Diagnostics) with tail-tip bleeding.

### Biochemical analysis

Glycogen content was determined in samples of frozen tissue by measuring amyloglucosidase-released glucose from glycogen as previously described (59). The intracellular concentration of ATP and AMP was measured by HPLC as previously described (55). Hepatic and muscle triacylglycerols (TAGs) were quantified in 3 mol/l KOH and 65% ethanol extracts as previously described (60). The intracellular concentration of glucose-6-phosphate (G6P) was measured in perchloric acid extracts with a fluorometric assay, as described (61).

### Western blot analysis

Liver samples were homogenized in 50 mM Tris/HCl (pH 7.4), 150 mM NaCl, 1 mM EDTA, 5 mM sodium pyrophosphate, 1 mM sodium orthovanadate, 50 mM NaF, 1% NP-40, 1 mM PMSF, and a protease inhibitor cocktail tablet (Roche). Immunoblot analysis of homogenates was performed using the following antibodies: phosphoenolpyruvate carboxykinase (PEPCK) (a kind gift from Dr E. Beale) at a dilution of 1:100.000. Proteins were detected by the ECL method (Immobilon Western Chemiluminescent HRP Substrate, Millipore, Sigma-Aldrich). The loading control of the WB membrane was performed using the REVERT total protein stain.

### RNA extraction and quantitative RT-PCR

Liver and muscle RNA extraction, RT-PCR, and quantitative real-time PCR analysis were performed as previously described (62). The following TaqMan probes (Applied Biosystems) were used for quantitative real-time PCR: *Ptg* (Mm01204084_m1), *Gys2* (Mm00523953_m1), *Pygl* (Mm01289790_m1), *Gbe-1* (Mm00472359_m1), *Pgm2* (Mm00728285_s1), *Gk* (Mm00439129_m1), *Pepck* (Mm00440636_m1), *G6pase* (Mm00839363_m1), *Pdk4* (Mm01166879_m1), *Pgc1α* (Mm00447180_m1), *Nr4a3* (Mm00450071_g1), *Ucp3* (Mm00494077_m1), *Murf1* (Mm01185221_m1), *Hk2* (Mm00443385_m1), and 18S (Mm03928990_g1). 18S was used as a housekeeping gene.

### Statistical analysis

Data are expressed as the mean ± SEM. *p* Values were calculated using two-way ANOVA with post hoc Tukey’s test as appropriate or two-tailed *t* test.

## Data availability

The data are available on request from the corresponding author (Iliana López-Soldado, IRB Barcelona, iliana.lopez@irbbarcelona.org).

## Supporting information

This article contains supporting information.

## Conflict of interest

The authors declare that they have no conflicts of interest with the contents of this article.
