# Supplementary material for: Increased liver glycogen levels enhance exercise capacity in mice
Source: J Biol Chem. 2021 Jul 18;297(2):100976. doi: 10.1016/j.jbc.2021.100976 (PMC8350413; doi:10.1016/j.jbc.2021.100976)
Supplement: Supplemental Figures S1 and S2 [file mmc1.pdf]

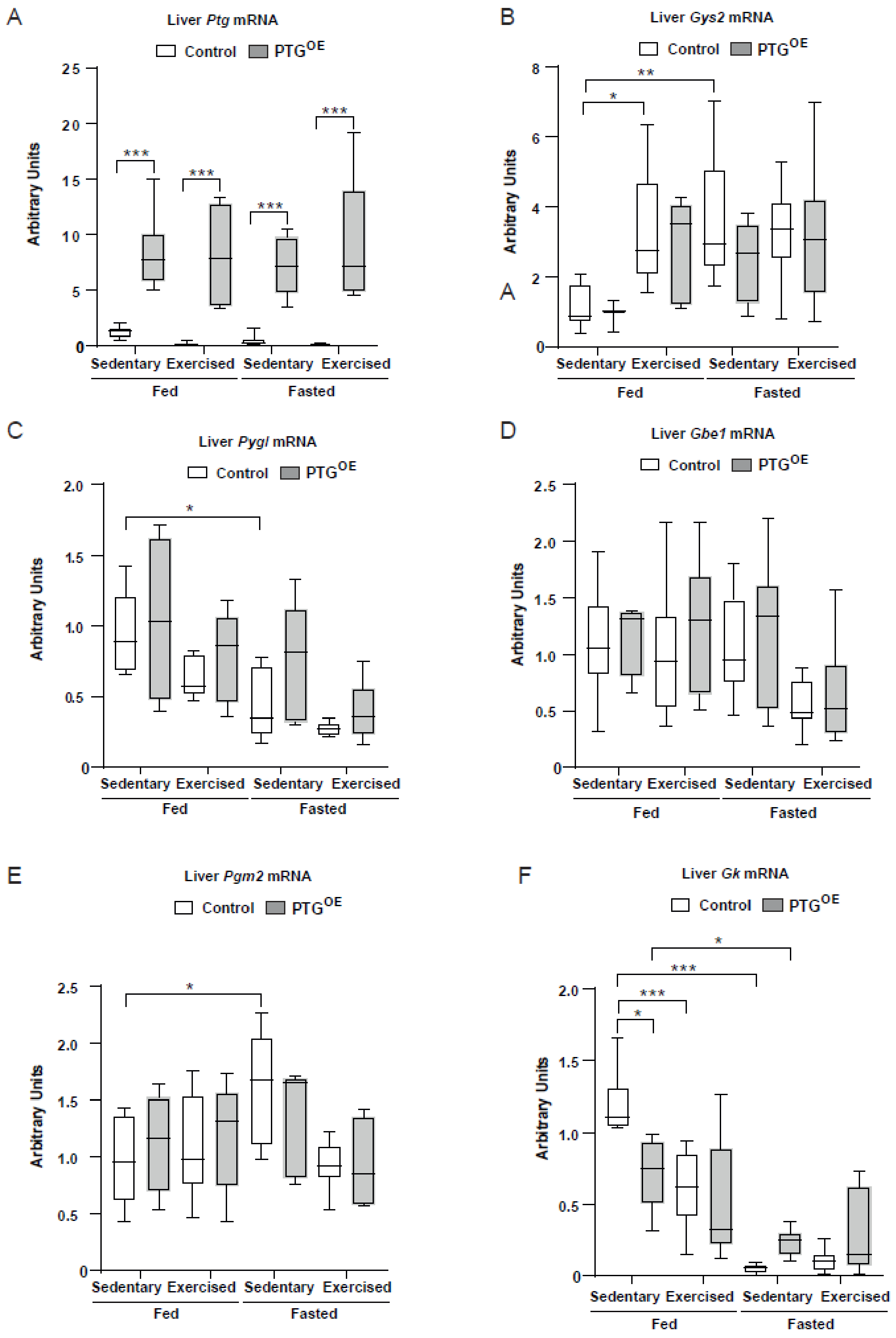

Supplementary Figure 1: Hepatic gene expression. (A) *Ptg* mRNA expression, (B) Gys2 mRNA expression, (C) *Pygl* mRNA expression, (D) *Gbe1* mRNA expression, (E) *Pgm2* mRNA expression, and (F) *Gk* mRNA expression in sedentary and exercised control and PTG^{OE} mice under fed and fasting conditions (n=8-12 in all experiments). All values are mean ± SEM. * P<0.05, ** P<0.01, *** P<0.001.

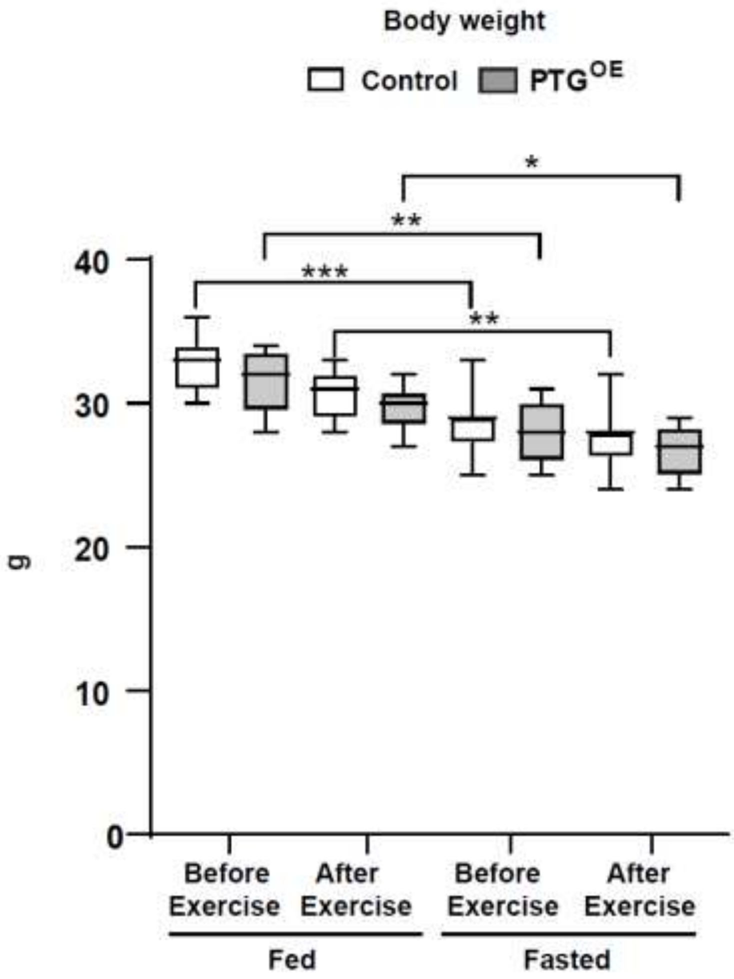

Supplementary Figure 2: (A) Body weight in control and PTG^{OE} mice under fed and fasting conditions before and after exercise (n=8-12 in all experiments). All values are mean ± SEM. * P<0.05, ** P<0.01, *** P<0.001.
